# Therapy-Related Myeloid Malignancies in Myeloma

**DOI:** 10.4084/MJHID.2011.047

**Published:** 2011-10-24

**Authors:** X. Papanikolaou, B. Barlogie, S.Z. Usmani

**Affiliations:** Myeloma Institute for Research & Therapy, University of Arkansas for Medical Sciences, Little Rock, AR

## Abstract

Therapy related myeloid malignancies are an increasingly recognized treatment complication in patients undergoing therapy for multiple myeloma. The main predisposing factors are the alkylating agents, topoisomerase II inhibitors and radiotherapy, but recently questions have been raised regarding the immunomodulatory agent lenalidomide. Little is known about the new antimyeloma agents in the context of therapy related myeloid malignancies. The duration of treatment and the time from diagnosis are the main contributing factors in alkylating induced myeloid malignancies which occur 5–10 years after treatment, chromosome 5 and 7 abnormalities being the characteristic finding. High dose therapy (HDT) does not seem to be a major contributing factor *per se* in multiple myeloma. In a number of large published series, all the factors related with therapy-induced myelodysplasia were defined prior to HDT. Topoisomerase II inhibitors induce mainly acute leukemias which invariably correlate with dysregulation of the MLL gene. Radiotherapy causes therapy related myelodysplasia if applied in bone marrow producing areas, especially if combined with chemotherapy. Therapy related myeloid malignancies generally herald a poor prognosis. Karyotypic abnormalities seem to be the main prognostic factor. In all cases the risk for therapy related myeloid malignancies drops sharply by 10 years after the treatment.

## Introduction

The problem of therapy related myelodysplastic syndromes (t-MDS) and acute myeloid leukemia (t-AML) in the context of cytotoxic chemotherapy is perhaps as old as the cytotoxic chemotherapy itself^1^ and it is part of the more general problem of the second malignancies after cytotoxic chemotherapy.^2^ It is a well known fact that as the overall survival (OS) for a malignant disease increases due to the treatment done, so the late effects of this treatment become much more evident with the advent of time.^3^

Multiple Myeloma (MM) is the second most prevalent hematological malignancy in the Western World^4^ the last 15 years. Since the beginning of the era of chemotherapeutical agents in the 70’s where the rate of complete remission (CR) for MM was below 3%, with the incorporation of tandem autologous hematopoietic cell –supported high dose therapy (HDT)^5^,^6^ and the newer agents as thalidomide, lenalidomide and bortezomib, the rate of CR has increased to over 80% under the Total Therapy TT3 protocol,^7^ making the MM median OS well over the past three year landmark. In fact 10-year survivals of over 30% have been observed.^7^ It is thus a natural consequence that the problem of t-MDS and t-AML becomes significant, requiring more attention form a biological perspective and likely requires special therpuetic considerations.

## Epidemiology

It is rather appropriate that if one considers and examines the epidemiological data of t-MDS in MM to firstly acknowledge the fact that MDS and MM can co-exist *de novo*. Both the morphological^8^ and the cytogenetic^9^ evidence of this fact have been well described, with the cytogenetic anomalies seen in ~4% of the total MM patient population and having a distinctly different prognosis from the rest of the MM subtypes.^9^ In a series of 648 MM patients that were enrolled in two non-HDT British Medical Research Council trials,^10^ the 5-year actuarial prevalence and the 8-year prevalence of t-MDS were 3% and 10% respectively (FAB morphological criteria were used for t-MDS and t-AML diagnosis). This series brought to the forefront, the issue of MM-therapy related myeloid neoplasms, a fact that was preciously well known in the context of other hematologic malignancies. The Arkansas group reported on the cytogenetically defined MDS of more than 3000 MM patients that underwent HDT^11^ and reported a prevalence of cytogenetically defined MDS of 3%. Most of the cytogenetic abnormalities (68%) were transient and clinical t-MDS and t-AML developed in 26 patients. It is therefore evident that there is a discrepancy of the reported prevalence and incidence of t-MDS in the various big series of the MM patients. Given the available knowledge on the main causes of t-MDS, one has to evaluate the incidence of MDS in the context of the therapeutic regimen given.

## Conventional Chemotherapy and t-MDS in MM

The causative relationship of alkylating therapy in MM and t-MDS has been acknowledged as early as in the 1970s.^13^,^14^ The widespread use of alkylating agents in various hematological and non hematological malignancies has resulted in valuable knowledge of the characteristics of the alkylating induced t-MDS. It is occurring mainly as a late event of the chemotherapy with a characteristic latency of 5–10 years.^15^ Patients will present with t-MDS and evidence of bone marrow failure with at least one cytopenias while a minority will present as t-AML or t-myeloproliferative/myelodysplastic syndrome.^16^ This category is commonly associated with unbalanced loss of genetic material, often involving chromosomes 5 and/or 7, although that is not universal.^16^ The decades of therapeutic experience has also contributed the knowledge that it is the amount of time and cumulative dosing of these agents and not the intensity of the therapy that contributes to the development of t-MDS. This fact is well established in many malignancies^17^ and is also evident in the MM population.^10^ Also well established is the knowledge that all alkylating agents are not the same in their leukaemogenic potential. Melphalan and BCNU are considered more leukaemogenic than cyclophosphamide in general^18^ and this fact has also been established also in MM patients treated with these drugs.^10^ The combination of alkylating agents and radiotherapy increases the incidence of t-MDS.^16^

The second category of t-MDS related to the conventional chemotherapy is related to the topoisomerase II inhibitors, namely adriamycin, etoposide, chemotherapeutics that interact through DNA topoisomerase II. This category of chemotherapeutics has long been successfully used in the treatment of MM. The t-MDS/AML that they produce has a latency period of 1–5 years, usually does not present as a t-MDS but as an overt t-AML from the beginning and is often associated with balanced chromosomal translocation.^16^,^17^ The amount of cumulative dosage is equivocal and in the setting of the therapy of other hematological malignancies several regimen-related factors, as the schedule and concurrent use of asparaginase and G-SCF, are important in determining the relative risk.^18^,^19^ Especially etoposide has strictly been associated with translocations of the MLL gene on chromosome band 11q23. MLL is a critical transcription regulator and the fact that there are over 40 partner genes in reciprocal translocations found in MDS/AML, suggests that it holds a crucial role in the pathogenesis of t-MDS/AML and MDS/AML in general.

In practice however most MM patients have received polychemotherapy of the above substances/modalities either concurrently or subsequently. The boundaries of the chromosomal, clinical and laboratory characteristics of the resulting t-MDS/AML characteristics regarding the causal chemotherapeutic are not always sharp.^11^

## t-MDS/AML and HDT in MM

HDT has become the standard of care in the management of younger patients with symptomatic or progressive MM.^20^,^21^ Tandem autotransplantation has doubled survival in relationship to standard-dose therapy.^22^ Sizable series have reported on the development of t-MDS/AML in the context of HDT for Hodgkin, non-Hodgkin lymphoma as well as MM.^11^,^23^,^24^ There is a clear tendency for attribution of t-MDS/AML, at least in the Hodgkin and non-Hodgkin lymphomas, in HDT. Since standard dose regimens precede autologous peripheral blood stem cell (PBSC) collection and HDT, it is unclear whether the t-MDS is associated with HDT or the preceding chemotherapy. Primary HDT after non stem cell damaging vincristine – adriamycin-dexamethasone (VAD) therapy resulted in an incidence of t-MDS at 0% at 4.7 years.^25^ In the biggest HDT MM series reported till now^11^ multivariate analysis showed that the t-MDS/AML development was correlated with age −15% in 10 years for the older patients (>65 years), poor (<2.5× 10^6^/kg) PBSC collection, time interval between the preceding chemotherapy and HDT reflecting longer pre transplant chemotherapeutic exposure and low platelet recovery 3 months after the first transplantation (<150×10^9^/kg). The type of the HDT regimen was not significant in terms of subsequent t-MDS/AML development. From the aforementioned it appears that HDT is likely a contributing factor in t-MDS/AML, along with a host of other important ones. The later is supported from the fact that studies in lymphoma patients that applied fluorescence in situ hybridization (FISH) analysis for the detection of MDS lesions in interphase cells, found that such abnormalities were already present in PBSCs prior to HDT and were similar or identical to those subsequently detected after HDT.^26^ Thus the question of the main contributing factor remains still open and could very well be that the main contribution of HDT to t-MDS/AML in MM is improvement in overall survival and patient longevity.

## Newer Therapies and t-MDS in MM

Very little is known about the contribution or not to t-MDS of the newer MM therapies. There were not differences in the incidence of t-MDS between the thalidomide and control arm in the Arkansas Total Therapy 2 trial.^11^ The recent reports on the association of lenalidomide with myeloid malignancies have born mixed results. The IFM 2005-02 study^27^ and CALGB 100104 study^28^ reported increased incidence of second primary malignancies, including myeloid malignanices, in the order of 5.5%–6.5%. In the MM-015 study, Palumbo et al^29^ reported a 0.7% incidence of t-AML/t-MDS in MM transplant ineligible patients with use of lenalidomide combined with melphalan/prednisone and receiving additional lenalidomide maintenance, compared with those receiving melphalan/prednisone alone. This has given rise to the debate of optimal duration of maintenance with lenalidomide, as it clearly has shown progression free survival benefit in MM. To date, there have no reports regarding bortezomib in t-MDS/t-AML development in MM or lymphoma patients.

## Therapeutic Modalities and Future Directions

It is crucial for anyone to realize that preventing is far better than treating! Present and future efforts have to be -at least partially- directed towards the maximum effective anti-MM therapy with the lowest t-MDS potential. For conventional chemotherapy cumulative experience favors the short exposure to alkylating agents without intensity of treatment being a worrying factor in terms of t-MDS development. Radiotherapy perhaps should better be avoided upfront and concurrently with chemotherapy at least in bone marrow producing regions. There are enough data to support its leukaemogenic potential but not enough data to support its superiority in MM treatment at least compared with other therapeutic modalities. Bortezomib and thalidomide seem rather safe agents in MM regarding t-MDS.^30^ The role of lenalidomide in t-MDS in the context of maintenance treatment in MM seems rather controversial. There is a clear need for more series with the maximum amount of uniformity for the rest of the MM treatment for someone to draw more definite conclusions.

Drug or xenobiotic metabolizing enzymes (DME) play central roles in the metabolism, biotransformation, and detoxification of xenobiotics and foreign compounds. They generally protect from potential harmful insults from the environment and also influence the metabolism of drugs (Table 1). Polymorphisms of these genes have been associated with the development of t-MDS/AML relative to the previous cytotoxic therapy. Although some of the reports are conflicting, the hall concept appears to be a very promising sector of pharmacogenomics and the individualization of cytotoxic therapy in general.^31^

The prognosis of t-MDS/AML is generally considered poor. An overall 5-year survival of less than 10% is commonly reported.^15^ It is strongly associated with the underlying karyotypic abnormality, something that recently has been recognized in *de novo* MDS also, as it is portrayed in the revised IPSS that showed in the last International MDS Symposium (ISMDS 2011, Edinburgh May 18–21). Cases with abnormalities of chromosome 5 and/or 7 and a complex caryotype have a particular poor prognosis with a median survival of less than one year regardless of the number of myeloblasts present in bone marrow biopsy at initial MDS diagnosis.^32^,^33^ Perhaps for these patients, an allogeneic transplantation should be strongly considered upfront. For not eligible patients autologous transplantation with PBSC collected early in the course of the patient could serve as an alternative. In the cases of 5q- chromosomal abnormalities lenalidomide has proved a valuable drug in releaving the accompanying anemia and in some cases inducing cytogenetic remission.^34^ The drug can be given also to non 5q-MDS with a amount of myeloblasts <10% with good results as long as the Gene Expression Profile of the MDS resembles the one of 5q- syndrome.^34^ Hypo ethylating agents azacytidine and decitabine although have promising results in *de novo* high IPSS MDS, have not been tested enough in t-MDS/AML and the results in cases with 7 monosomy and complex caryotype are rather disappointing. Perhaps their use is better suited for cases of t-MDS with a number of myeloblasts>10% and karyotypic abnormalities that represent balanced translocations. This group can also benefit from the traditional chemotherapy approach at least for induction and/or salvage chemotherapy in terms of RAEB II MDS or t-AML. Of notice is the fact that the rare antracycline related Acute Promyelocytic Leukemias herald the exact same prognosis with the *de novo* ones,^35^ a fact that highlights the importance of the underlying karyotypic abnormality in the prognostic and therapeutical evaluation of t-MDS. Supportive care (erythropoietin agents, transfusion policy, iron chelating therapy) is the same as with the *de novo* MDS.

## Conclusions

t-MDS represents a real and emerging problem in MM treatment. As the median MM OS survival universally increases it will possibly establish further its presence in the MM course. Although the diagnostic, prognostic and therapeutic capabilities of t-MDS and MDS in general are continuously expanding, one has to remember that “to prevent is always better than curing” meaning that a good amount of present and future efforts has to be concentrated in the recognition and improvement of the MM therapy with the best anti myeloma effect and the fewer t-MDS complications.

## Figures and Tables

**Table 1:** Role of gene polymorphism in t-MDS/t-AML development

Class		Glutathine-S Transferase pathway(GST)	Cytochorome P 450 system (CYP)	DNA Repair system
**Alkylating Agents**	Busulfan BCNU Cyclophosphamide Mechlorethamine Melphalan	GSTM1 GSTP1 GSTT1	CYP2B6 CYP2C19 CYP3A4	MGMT1 BER RAD51 XRCC3
**Topoisomerase I Inhibitors**	Irinotecan Topotecan		CYP3A	NHEJ (Non-homologous end joining)
**Topoisomerase II Inhibitors**	Daunorubicin Doxorubicin Etoposide Mitoxantrone Teniposide	GSTP1	CYP1B1 CYP3A4	NHEJ (Non-homologous end joining) RD51 XRCC3 NQ01
**Ionizing Radiation**				RD51 XRCC3 NQ01
